# Low Levels of Awareness Despite High Prevalence of Schistosomiasis among Communities in Nyalenda Informal Settlement, Kisumu City, Western Kenya

**DOI:** 10.1371/journal.pntd.0002784

**Published:** 2014-04-03

**Authors:** Gladys O. Odhiambo, Rosemary M. Musuva, Vincent O. Atuncha, Elizabeth T. Mutete, Maurice R. Odiere, Rosebella O. Onyango, Jane A. Alaii, Pauline N. M. Mwinzi

**Affiliations:** 1 Centre for Global Health Research, Kenya Medical Research Institute, Kisumu, Kenya; 2 School of Public Health and Community Development, Maseno University, Maseno, Kenya; 3 ContextFACTOR Solutions, Nairobi, Kenya; Ministry of Health, Uganda

## Abstract

**Introduction:**

Intestinal schistosomiasis is widely distributed around Lake Victoria in Kenya where about 16 million people in 56 districts are at risk of the infection with over 9.1 million infected. Its existence in rural settings has been extensively studied compared to urban settings where there is limited information about the disease coupled with low level of awareness. This study therefore assessed community awareness on existence, signs and symptoms, causes, transmission, control and risk factors for contracting schistosomiasis as well as attitudes, health seeking behaviour and environmental antecedents that affect its control so as to identify knowledge gaps that need to be addressed in order to strengthen schistosomiasis control interventions in informal urban settings.

**Methods:**

The study was carried out in an informal urban settlement where the prevalence of intestinal schistosomiasis was previously reported to be the highest (36%) among the eight informal settlements of Kisumu city. The study adopted cross-sectional design and purposive sampling technique. Eight focus group discussions were conducted with adult community members and eight key informant interviews with opinion leaders. Data was audio recorded transcribed, coded and thematically analyzed using ATLAS.ti version 6 software.

**Results:**

Most respondents stated having heard about schistosomiasis but very few had the correct knowledge of signs and symptoms, causes, transmission and control of schistosomiasis. However, there was moderate knowledge of risk factors and at high risk groups. Their attitudes towards schistosomiasis and its control were generally indifferent with a general belief that they had no control over their environmental circumstances to reduce transmission.

**Discussion/Conclusion:**

Although schistosomiasis was prevalent in the study area, majority of the people in the community had low awareness. This study, therefore, stresses the need for health education to raise community's awareness on schistosomiasis in such settings in order to augment prevention, control and elimination efforts.

## Author Summary

Bilharzia also known as schistosomiasis is one of the neglected tropical diseases found in western part of Kenya. The major source of infection is Lake Victoria; however, there is evidence of inland transmission especially within the informal settlements of Kisumu city. Schistosomiasis can be controlled using three key approaches which include improved sanitation, health education and mass treatment with praziquantel. Additional interventions for infection prevention include: promotion of hygiene, access to safe water, and sanitation improvement and environmental management. However, the success of control initiatives involving the community depend on the level of the communities' uptake of the program, which is hinged upon understanding the community knowledge and practices towards the disease. This study therefore collected information from the community to assess level of awareness of schistosomiasis. The findings revealed a low level of awareness in spite of a high prevalence of schistosomiasis. These findings are invaluable in the designing of appropriate education messages targeted at raising community awareness on schistosomiasis and relevant behavioural change required for a successful control programme.

## Introduction

Schistosomiasis is one of the neglected tropical diseases, found in tropical and subtropical areas of Africa, Asia and Latin America [1] where it is found in both urban and rural settings where sanitation and control efforts are inadequate and populations are impoverished, thereby considered a “poor man's disease” [2]. Worldwide, about 207 million people in 74 countries are infected with schistosomiasis, with sub-Saharan Africa (SSA) accounting for 93% of cases [3]. In Kenya, prevalence of the disease ranges from 5% to over 65% in different communities and contributes to significant morbidity [4], [5], [6], [7]. It is also estimated that 16 million people in 56 out of 158 districts are at risk of the disease [8] and over 9.1 million are infected in the country [9].

Schistosomiasis is majorly reported for rural settings, while urban settings are often not considered as transmission foci, resulting in limited evidence on the disease and subsequent neglect of urban areas [10]. Schistosomiasis transmission in urban settings may result from contamination by infected migrants in search of employment, and the poor sanitation around freshwater harboring snail intermediate hosts. In Kenya, rapid urbanization amid economic downturn has resulted in increased proportions of people living in absolute poverty in the urban areas [11], including informal settlements of cities such as Kisumu where overcrowding and inadequate water and sanitation are a major challenge [12]. Moreover, in informal settlements, the low quality of housing and the general lack of basic infrastructure especially sanitation drainage, access to energy and clean water supply result in poor social and environmental conditions like poor living conditions and low level of education [13] which have significant impact on the spread of infectious diseases, including promotion of transmission of schistosomiasis.

Schistosomiasis can be controlled using three key approaches which include improved sanitation, health education and mass treatment with praziquantel. The government of Kenya is now providing free treatment of schistosomiasis and soil transmitted helminths to school age children but the adults in the community who are equally vulnerable can barely obtain treatment which is not only inaccessible but also unavailable to them. Promotions of hygiene, access to safe water, and sanitation improvement as well as environmental management are additional interventions for infection prevention. Transmission of infection can also be controlled by targeting snail vectors and avoiding contact with infected waters [1]. However, the success of control initiatives involving the community depend on level of the communities' uptake of the program, which is hinged upon understanding the community knowledge and practices towards the disease and recommended preventive and/or treatment regimens. Indeed, the Kenyan Ministry of Public Health and Sanitation (MOPHS) through its national multi-year strategic plan for control of Neglected Tropical Diseases 2011–2015 also recommends that more research is needed on the knowledge, attitudes and practices towards schistosomiasis control [8]. This study aimed at assessing community knowledge, attitudes and practices on schistosomiasis, (its existence, signs and symptoms, causes, transmission, control and risk factors), the findings of which will inform bridging of identified gaps to enhance a successful control programmes in Nyalenda B, an informal settlement in Kisumu city, western Kenya.

## Materials and Methods

### Ethics statement

This study was approved by the Scientific Steering and the National Ethics Review Committee of the Kenya Medical Research Institute (KEMRI, SSC # 1841). Permission was obtained from the Provincial Commissioner, Nyanza, the Director of Public Health and Sanitation Nyanza Province, the Town Clerk of Kisumu City and the Municipal Public Health Officer of Kisumu City. Permission was then sought from the area assistant chief and village elders were notified about the study. All participants gave written informed consent. All information given by the study participants were kept confidential and anonymity was highly observed. No personal identifiers were used during data entry and analysis.

### Study site, population and healthcare delivery

The study was conducted in Nyalenda ‘B’ sub-location which is an informal settlement located in Kisumu city, western Kenya. The sub-location covers an area of 6.1 Km^2^ with a total population of 32,430 people, 16,189 of whom are male and 16,241 female [14]. The sub-location is headed by an Assistant Chief. There are five units which are further sub-divided into nine sub-units within the sub-location. These sub-units are headed by one or two village elders depending on size. The informal settlement has four municipal-run primary schools (Joel Omino, St. Vitalis Nanga, Pandpieri and Dunga) that have been greatly overwhelmed by increased enrolment since introduction of universal free primary education in the country [12]. The main economic activities in the area include fishing, car washing and small business enterprises.

Intestinal schistosomiasis is a public health problem in the area and its prevalence of 36% is highest compared to all the other informal settlements in the city [10]. Presence of infected intermediate host snails has also been reported, suggesting active transmission [15].

Poor sanitation prevails with high use of bush or fly toilets [12] and domestic water sources ranging from taps, springs, boreholes, water vendors to Lake Victoria.. The area is served by only one frontline government health facility; but there exist a few private clinics and a private hospital.

### Study design

The study was a cross-sectional, descriptive assessment that employed qualitative methods, including focus group discussions (FGDs) and key informant interviews (KIIs). FGDs were conducted with community members to gauge community knowledge, attitudes and practices towards schistosomiasis control. KIIs were conducted on opinion leaders on schistosomiasis its control in terms of water, sanitation and treatment. The KIIs were conducted before the FGDs in order to get views of opinion leaders about schistosomiasis to facilitate focused questions to understand the feelings of the general community members, thus both were used to complement each other. After the tools were developed, they were piloted in an informal setting with similar characteristics as Nyalenda B where we carried out the study. Since generally 4 focus groups are considered adequate for a given research question with a given group of target study participants [16], we conducted eight FGDs so that each category is represented by two so as to minimize bias of chance.

The data was collected by trained KEMRI (Kenya Medical Research Institute) personnel using both audio recorders and field notes. Eight FGDs, each comprising 10–12 participants, and 8 KIIs were conducted on purposively selected respondents. The total number of participants was 88. Four of the 8 FGDs comprised of unmarried youth adults (18–24 years of age) while the remaining four comprised of ever married persons (>24 years of age). The FGDs were conducted separately for different sexes and age groups (Table 1). KII participants were either married or widowed (Table 2).

**Table 1:** Socio-demographic characteristics of the study population in focus group discussions.

		Ever Married Adults		Unmarried youthful Persons	
Variable		Male	Female	Male	Female
	Number of Participants	20	20	22	18
Age	Mean[range]	42 [30–63]	36 [25–48]	22 [19–24]	20[18–24]
Marital Status	Living with another	0	0	2(9)	3 (17)
	Single	0	0	20(91)	15(83)
	Married	17 (85)	15 (75)	0	0
	Widowed	0	5 (25)	0	0
	Divorced/separated	3(15)	0	0	0
Education (%)	None	0	0	0	0
	Primary	4 (20)	5 (25)	0	0
	Secondary	6 (30)	13(65)	2 (9)	6(33)
	Post Secondary	10 (50)	2(10)	20 (91)	12(67)
Occupation (%)	Subsistence farming	1 (5)	1 (5)	0	0
	Skilled labour	6 (30)	3 (15)	4(18)	3(17)
	Unskilled labour	1(5)	1(5)	2(9)	
	Business owner	6 (30)	8 (40)	2 (9)	6(33)
	Student	0	0	10 (45)	5(28)
	Unemployed	3(15)	5(25)	1 (5)	3(17)
	Fishing/Fish Monger	2(10)	2(10)	2(9)	0
	Salaried worker	1(5)	0	0	1(6)
	Other	0	0	1(5)	0

**Table 2:** Socio-demographic characteristics of key informants.

Variable		Male	Female
	Number of Participants	3	5
Age	Median[range]	52 [38–59]	45 [30–54]
Marital Status	Married	2 (67)	3 (60)
	Widowed	1(33)	2 (40)
Education (%)	None	0	0
	Primary	1 (33.3)	1 (20)
	Secondary	1 (33.3)	3(60)
	Post Secondary	1 (33.3)	1(20)
Expertise (%)	Group Leader	2(67)	3 (43)
	CHW	0	2 (29)
	Church Leader	0	1 (14)
	Farmer	0	1 (14)
	Village elder	1(33)	0

Permission for the study was obtained from the local administration following briefing on the study. The study team was then introduced to the village elders who assisted in mobilizing adult household members from the various sub-units ensuring representation of at least one participant in each FGD group. The participants were screened for eligibility before the discussion commenced to avoid selection bias. The criteria was that one must have lived in the study area for more than six months, be an adult and able to articulate their speech bearing in mind the representation from all the subunits. The KII were conducted with one opinion leader from each sub unit. The FGDs lasted one and half hours and the KII took about one hour. Socio-demographic profile questionnaire was administered to all the participants.

### Data management and analysis

All the qualitative data collected was transcribed verbatim and the text typed into the computer. The data cleaning and analysis was done using ATLAS.ti version 6. A code sheet was created following the focus group and the key informant guides after which, the textual data was coded into selected themes and a master sheet analysis was carried out, giving all the responses from the FGDs and KIIs a theme. Thematic analysis [17] was used where responses were categorized into themes and then ideas formulated by looking at the patterns of responses. Analyzed data were presented in text form. Quantitative data from the socio-demographic profiles was analyzed using excel spreadsheets.

## Results

### Knowledge and level of awareness

#### Awareness about local name, existence as well as signs and symptoms of schistosomiasis

Various local names for schistosomiasis were given as: *Nyanam, Layo remo, Ndira* (also used to refer to cholera) *and Aremo*. *Nyanam* and *Aremo* were the most commonly used names. Even though most of the participants declared having heard about schistosomiasis before, especially during schooling, generally the level of awareness was low. Many of those who reported to have heard about schistosomiasis thought that it is airborne, did not know its correct signs and symptoms, cure and prevention. Some also stated that they had never heard about the disease at all.

*“…community does not know, there is lack of awareness on what you can really do to know that you have bilharzia”* **Adult female (FGD).**

*“And for bilharzia we should give it a priority when it comes to awareness, because, not many people know bilharzia…”* **(KII).**

Some participants knew the correct signs of schistosomiasis- which they associated with blood in either stool or urine, but, majority did not know the correct signs as illustrated below:

*“……. another symptom is when someone is having cracks in the feet”* **Adult female (FGD).**

*“When you look at the eyes of the person apart from the normal it is normally white, because they physically have the bilharzia it is yellow…… And in extreme condition they get to malaria.”* **Adult male (FGD).**

#### Awareness on causes and transmission

There were various causes of schistosomiasis mentioned by the participants. Majority thought that the disease was caused by drinking contaminated or untreated water. Lack of toilet was also considered another cause.

*“… we normally go to the beach and we do swimming there…..I go for long call… then another person, [comes to] fetch the water and take in to the house to drink without boiling….he or she will be also affected.”* **Adult female (FGD).**

Some of the participants knew that snails and poor sanitation contributed to spread of the disease, but lacked understanding of the transmission cycle.

*“Another step is like sometime you can take something like a sugar cane but you dint* [did not] *wash it because the time the sugar cane is there* [in the farm], *things like the snails is just walking around… when you take it when you have not washed you will get bilharzia……….”* **Adult male (FGD).**

Taking contaminated food, walking barefooted, sharing of bathroom and open defecation were also perceived to be other causes of schistosomiasis.

#### Groups at risk

Generally everyone was perceived to be at risk, however, children were perceived to be most at risk followed by fishermen, poor families and people living along the shores of the lake, in that order.

*“Children are at a high risk because they run barefooted. Sometimes we give them shoes to put on like slippers but they will run off…When they feel thirsty they can even drink anything around.”* **Adult female (FGD).**

*“Fishermen stay in water several hours; several hours in water and even the boats they use, underneath those boats are hosts of snails, the nets they use they catch the snails. They work with snails entirely”* **Male youth (FGD)**.

#### Awareness about control and prevention measures

Participants mentioned various ways of curing schistosomiasis, these included: treatment, buying drugs from Community Health Workers/pharmacy, prayers and herbal remedies. Participants also highlighted that use of protected wells, application of snail insecticides, avoiding contact with stagnant water, wearing shoes, health education would also help in combating the infection. However some respondents thought that one could prevent the disease by lowering the rate of deforestation. Moreover, more than half of the respondents also thought that drinking treated water was a good control strategy (Text Box 1).

**Box 1. Awareness on Control and Prevention Measures**

**By treatment or buying drugs from the medicine shops**

*“Some buy drugs from the shops… people don't know Bilharzia is there, so sometimes you have Bilharzia, but you are fighting treating typhoid…”* **Adult female (FGD).**

**By prayers**

*“Okay there are some churches they believe that they don't go to the hospital at all and they go for prayer. So they believe that if you have strong faith, your faith will heal you, and they get healed, then later it can come back again but they believe it cures.”* **Adult female (FGD).**

**By herbal remedies**

*“… Some people here just believe in treatment with herbs so they treat bilharzia using it. They drink the herbs from witchdoctor”* **Adult male (FGD).**

**Proper disposal of waste and dirty water**

*“… So to avoid that we must have drainage and the latrines must be kept well.”* **Adult male (FGD).**

**Creating awareness and drinking safe water**

*“We should create more awareness to community members and also our family… so that we get more info (information) about it. And then, take more precaution by drinking safe water.”* **Adult female (FGD).**

**Snail Control**

*“… and application of insecticides particularly to those areas infested with snails.”* **Adult male (FGD).**

**Development**

*“…when we have a bridge that crosses the river, people won't step on the water… If development is improved and infrastructure is improved, I think Bilharzia won't be a problem.”* **Female youth (FGD).**

**Hand washing and dish racks**

*“And another thing, there is a campaign for washing hands after going to the toilet and before eating…. So that is another campaign that I think reduces the transmission of Bilharzia.”* **Adult male (FGD).**

Even though the participants felt that schistosomiasis was curable they mentioned a number of challenges which included lack of drugs in the hospital, lack of money and lack of diagnostic kits.

*“Me I would say that it is not very much effective because of lack of drugs and lack of money. If you go to the hospital and you do not have money they will just look at you there”* **Adult male (FGD).**

*“…the testing kit for bilharzia is also missing. They test malaria instead of testing bilharzia they don't have something to test it with.”* **Male youth (FGD)**.

On the other hand lack of alternatives and low socioeconomic status hindered prevention measures. These are illustrated below:

*“I think we can't just say we are going to avoid because that is where we are staying and we have to drink that water because we don't have another water. We have to go and fetch fish because that is where we are getting our income generating activity… If you are told not to go into the stagnant water, then what is the way out? … if we tell our men not to go and bring fish, then how will they eat?”* **Adult female (FGD).**

*“May be because my level* [my income], *my earning standard will only enable me to stay in that house where there is no toilet… rent rate now will be low because there isn't toilet.”* Key Informant Interview **(KII).**

The participants also stated that in some cases where the latrines were present, people still preferred the bush which they found more comfortable as compared to pits that were not only feared to house snakes but were also almost full in many cases.

According to them, there was not much they could do to control the infection. They said that their environment exposed them and since in most cases there was no alternative they ended up indulging in activities that continually exposed them to the infection (Text Box 2).

**Box 2. Environmental Antecedents**

**Lack of proper and relevant medication at the health facility is an environmental factor that influenced health seeking behaviour.**

*“In most areas you will find that drug prescription and diagnosis is not quite relevant to those visiting that hospital. If he is got malaria drugs he will base his diagnosis on that. Most people will be told that they have malaria since they only have malaria tabs there”* **Male youth (FGD).**

**The climate**

*“… There is always Bilharzia whenever we come to the rain seasons; we always have floods I don't know why? That is a failure. It is natural, the rain will come and the floods will come”* **Adult male (FGD).**

**Poverty**

*“….but it is because we are living below poverty line, sometimes somebody has the sketch of houses, which when the rain comes, the rain even sweeps the houses, then the next time the person does not have attention to dig another pit latrine. So this is why you get people are renting the houses and there is no latrine in that plot because we are living below poverty line…”* **Adult female (FGD).**

### Perceived severity of disease and attitudes towards interventions

Participants perceived schistosomiasis as a very serious disease which would cause even death.

*“Very serious and very contagious because once you have bilharzia you cannot go to bed with your wife. (Laughter) And then you cannot perform any activity that can give you income…. And then you grow thin and you grow weak, people loose appetite. People think you have AIDS while you do not have it. If you go to the hospital they test AIDS you do not have it you come back home you are still sick, you take painkillers, eventually you die. This is a sad scenario…”* **Adult male (FGD)**.

*“…we have bilharzia with us and it is a killer disease…”* Key Informant **(KII)**

Some of them had negative attitudes towards bilharzia treatment, its prevention and also towards those suffering from the infection (Text Box 3).

**Box 3. Attitudes towards Schistosomiasis**

Some had negative attitudes towards schistosomiasis treatment and Prevention

*“They are giving us generic drugs, so treatment is not effective”* **Youth male (FGD).**

*“It is not affordable, is not affordable that is why you will get flying toilet all over”* **(KII).**

Those perceived to be suffering from the infection were stigmatized

*“Yea because of the extended stomach some people would say they are cursed and would be just counting days for the death of that person”* **Adult female (FGD).**

*“There is stigma; those who have it are stigmatized in the society. People look at them like they are no more in life that they are dying.”* **Adult male (FGD).**

*“Some people … mistake the disease for HIV because of the extended stomach they mistake them and not take them to the hospital saying this is dying.”* **Male youth (FGD).**

*“Because of stigma. The fear that maybe if they go to the hospital the doctor how will he see me or how will he take me. So because of stigma they may not go.”* **Adult female (FGD).**

**Discrimination at the health facility**

*“… Some people don't prefer to go to the hospital. First, because of the way the doctors handle people nowadays in the hospital. You know us we believe you go to hospital when you are seriously sick and when you go to the hospital when you are seriously sick, the doctor will really discriminate you. He will say some sort of nonsense. What were you still doing? Why did you come late? You wanted to die? Just stay there.eeh they ignore you so when you think about that you say let me stay here if God can heal me or if this medicine I have taken from the pharmacy can heal me I can just stay…”* **Adult male (FGD).**

**Promiscuity blamed for infection**

*“My wife, because I have to ask her how she has been walking* [behaving] *and if I was true to her then she was not walking well* [unfaithful] *that is why she has brought the disease”* **Adult male (FGD).**

### Health-seeking behaviour

Community members highlighted that whenever they got sick they would seek some intervention. Going to hospitals, buying and taking pain killers were among remedies most mentioned.

*“….people would go for other alternatives, be it herbal, be it quack doctors, be it witch doctors and this is just to contain the situation. People would resort to go to bush doctors.”* **Adult male (FGD)**.

Perceived cost of medication appeared to influence behavior. Community members believed that medication for treating schistosomiasis was too expensive for them to access it. On the other hand perceived severity prompted people to seek medical attention; however, home management of schistosomiasis and other infections was majorly practiced.

*“They only prefer the facilities because of the severity, how severe is the disease…how it is severe then he is rushed to the hospital but in most cases they are home managed.”* **Adult male (FGD)**.

### Sources of information

The participants suggested that they would like to learn more about schistosomiasis in the following areas: signs and symptoms, prevention, cure, drugs, how long it takes before it kills someone, how it is spread, who are at risk and which hospital to go to for treatment. They suggested various means that information about schistosomiasis could be passed to them. These were: media, chief *barazas* (community meetings), church, funerals, posters, door-to-door campaigns, hospitals and over the radio.

*“I do suggest that door-to-door campaigns would do much good because ………..When someone come to your house and talk to you, you will come to understand that this thing is real.”* **Adult male (FGD)**.

*“We can use CHWs in the community so that they can mobilize, people knows them, and if they are mobilized and told there is something to be shared, the people will gather and then the facilitators come and give out the information”* **Adult female (FGD)**.

## Discussion

Regardless of age and gender the participants in this study had almost similar level of knowledge attitudes and practices on schistosomiasis control. However there was a general feeling that women were more concerned about health issues of their families than their male counterparts.

### Level of awareness

Our study found out that the overall level of awareness and knowledge about schistosomiasis amongst community members was low. This was similar to findings of a study done in Ethiopia [18]. In our study, drinking contaminated water was perceived to be the major cause of the infection thus avoiding it was considered a powerful prevention tool. This is in line with other studies [19], [20].

Poor sanitation, overcrowding, contact with contaminated water, community's level of knowledge, their attitudes and practices are factors that promote the transmission of schistosomiasis [21]. This study observed that community members still defecated in the open and had to go to the contaminated waters of Lake Victoria for various reasons even if they understood the dangers. Acka and others also reported a similar practice of poor hygiene, where, villagers tended to defecate where convenient – still rarely using latrines where available [22]. According to Rey [23], the habit of bathing after urination or defecation near picnic sites as a religious ritual contributed to maintenance of schistosomiasis transmission. This behavior was also observed by Farroq and Nallat [24].

This study revealed some of the misconceptions that community members have about schistosomiasis. People believe that those who had the infection were cursed because of the distended stomach, or were promiscuous because of blood in urine. However, this finding was not unique to our study. In Cameroon, residents of rural areas related hematuria to excessive exposure to sunlight and sexual intercourse thereby dismissing medical treatment in local hospitals as a result of such beliefs [25]. Stigma is known to increase feelings of fear, shame and reduces people's capabilities to successfully obtain appropriate treatment [26]. In our study those infected were stigmatized, people considered them “to be on their way to death” and if they were female they would be labeled promiscuous. However, they also appreciated that schistosomiasis could have other causes like drinking contaminated water which is a misconception.

Another study reported that it was shameful to have blood in urine [27], again highlighting social stigma that is associated with schistosomiasis.

### Attitudes

A study conducted in Kenya and Tanzania earlier found that schistosomiasis is considered a minor disease by many communities [28]. The disease was perceived not serious since it does not harm or prevent the victim from eating [29]. Our study found a contrary opinion. The community members perceived it to be a severe infection which would lead to death. We also found out that people would only resort to medical care if they realized that their condition was getting worse. Our other discoveries were similar to findings of another study which inferred that lack of money and disease not being serious enough made people not to go to hospital [30]. Moreover, in Ghana it was also established that perceived severity of the disease was the most important determinant of seeking health care or visiting a health facility [31].

In this study personal susceptibility was considerably high. Everyone deemed themselves at risk of getting schistosomiasis mainly because of the lake. This corresponds to a study in Uganda where the risk of infection was highest amongst those who lived near lakes or rivers [32]. However, children and fishermen were considered to be at the highest risk. Gender was not considered a major risk factor since some people thought that the women were more exposed because they do their domestic work at the lake or with water from the lake and others thought that it was men since they go fishing in the lake. Therefore the main risk factor was occupation and age. In this regard, it is worth pointing out that most community members are aware of the health risks of contact with Lake water, yet they lack the capacity to change their behavior, since they depend on the lake for subsistence.

### Practices

In our study, religious believes influenced health seeking behaviour. There were community members who would neither go to the hospital nor take medication and only believed in spiritual healing. Cost of medication also made community members stay away from seeking care since the perceived cost of treatment was considered too high by participants in our study. For this reason, the few who were perceived to be economically able were the ones who would afford health care according to our study. Likewise, in Cote d'Ivoire, people with higher socio-economic status more frequently sought health care and visited health facilities than people with low socio-economic status [33]. In many cases the disease was left untreated because people felt that they could not afford the treatment or that the drug was not available. Some people managed only the symptoms of the infection by using over-the-counter painkillers.

The finding that high cost of praziquantel (PZQ) and its unavailability in the hospital impacting negatively on the control of schistosomiasis was not unique to our study. This observation is comparable to a study in Nigeria where the progress in the control of schistosomiasis in the country had been insignificant due to high cost of PZQ [34]. Lack of PZQ in most peripheral health facilities in endemic areas of Ghana [35] also affected health seeking behaviour. Furthermore, of the health care facilities that would prescribe PZQ, only 60% had it in stock. The other reason that our participants gave for not going to the health facility to seek treatment, was that health facilities did not have diagnostic kits and the attitude of the healthcare workers was also discouraging. Other studies showed that health professionals in peripheral facilities referred patients for diagnostic test and/or treatment [30], [36].

There are people who tend to favor home-based treatment using various herbal remedies to treat cases of schistosomiasis and thus do not resort to medical treatment [37]. We also found a number of participants who only believed in herbal therapy. Whereas earlier studies showed a clear impact of distance on the utilization of health care facilities in rural Nigeria, where utilization declined exponentially with distance [38], our study showed that participants were not aware of any facility in their close proximity that offered schistosomiasis medication. They had to go to the referral hospitals where getting the drugs for the infection was not obvious and they are located far from their place of residence and therefore they must factor in transport costs. For this reason, and consistent with a study in Niger [39], people preferred traditional methods of treatment. Another study revealed that over 90% of those that self-medicated or visited chemical shops for treatment did not receive PZQ [30], consistent with our study where the community appreciated finding painkillers in the medicine shops.

In the environment there exists predisposing and enabling factors that influence people to behave the way they do. Just as observed in this study, cultural, socio-economic, geographical access and organizational - perceived quality (standard of drugs, standard of equipment, competence of staff, attitudes of staff, interpersonal process) are factors in the environment that can influence treatment seeking behaviour [40]. . Surveys performed in Egypt with young students and adults showed they had good information on schistosomiasis. Despite the fact that they knew they had to avoid being exposed to contaminated water, exposure was occurring for lack of other alternatives [28]. The same situation occurred in our study where people knew that to avoid contact with contaminated water was a healthy behaviour but could not uphold it due to dependency on the water for domestic and economic use including fishing, sand-harvesting and car washing. Participants indicated that there was nothing they could do about their situation because even if they put up latrines they are swept away by floods or they sink after a very short time and so instead of sinking in a latrine they would rather go to the bush or use “flying toilets” (rap and throw). This is in contrary to another study where people depended on the schistosomiasis-infected river for all the domestic needs even where there are alternative sources of water arguing that the river/stream gave them purer water than the hand dug well [29].

### Study limitation

The research instruments in this study were administered in English and Kiswahili which made the selection criteria biased towards only those who would speak in those two languages. However, this study area being cosmopolitan, these were the appropriate languages to use.

### Conclusions and recommendations

Outcomes of this KAP study can be used to design appropriate education messages, for which one needs to first understand what the gaps are in the knowledge that already exists. Health communications/education programs which form one of the social aspects of control should involve knowing the gap and intervening for it through understanding of perceptions, attitudes, and practices [41]. Beliefs about the threat of the disease (i.e. perceived severity and perceived susceptibility) would directly influence the likelihood of taking a recommended action (i.e. steps to control schistosomiasis). These perceptions could be affected by socio-demographic factors and knowledge. Perceived benefits and barriers may determine intended action, while cues to action could be information from others [42]. When disease control interventions are built upon lay knowledge and perceptions they become very effective [27].

This study showed that the level of awareness of schistosomiasis was low, people had negative attitudes towards control interventions and the practices documented only promoted the existence of the disease whereas environmental factors catalyzed its transmission. Although it may not be possible to avoid water contact activities and exposure to the parasite in the absence of other alternatives [28] especially for this community where their main economic activities revolve around the lake, it is believed that to raise community awareness of schistosomiasis as well as treatment-seeking behavior in such endemic areas, provision of health education is a useful strategy. This study, therefore, stresses the need for health education to raise community's awareness on schistosomiasis in order to strengthen the impact of control interventions. Such an education campaign should focus on causes, transmission, treatment and prevention of schistosomiasis. Governments are also urged to equip frontline health facilities with schistosomiasis diagnostic kits and the drugs (PZQ) in such endemic areas.

## Supporting Information

Checklist S1 STROBE checklist.
(DOC)

Text S1 Focus group discussion guide.
(DOC)

Text S2 Key informant interview guide.
(DOC)

Text S3 Social demographic profile.
(DOC)

Text S4 Consent form.
(DOC)
