# Supplementary material for: Low Levels of Awareness Despite High Prevalence of Schistosomiasis among Communities in Nyalenda Informal Settlement, Kisumu City, Western Kenya
Source: PLoS Negl Trop Dis. 2014 Apr 3;8(4):e2784. doi: 10.1371/journal.pntd.0002784 (PMC3974654; doi:10.1371/journal.pntd.0002784)
Supplement: Text S1 — Focus group discussion guide. (DOC) [file pntd.0002784.s002.doc]

**Supporting Text S1: Focus Group Discussion Guide**

**Title: Low Levels of Awareness despite High Prevalence of Schistosomiasis among Communities in Nyalenda Informal Settlement, Kisumu City, Western Kenya.**

**Who to interview:**  Residents of Nyalenda.

Good day. I am … … and my colleagues are …………. We are from …….. and we are here to learn from you about Bilharzia control in your community. The objective of this study is to collect information on the knowledge, attitude and practices on Bilharzia and its control in Nyalenda. The information you provide will be used to improve Bilharzia control. We have invited you because of your experience in this community and the confidence we have in you to be able to reflect the situation in Nyalenda. We will talk to you for about 30-45 minutes. Participation in this discussion is voluntary. Your name and what you say to us during this discussion will be kept confidential.

In this discussion, every answer within the study subject is correct, because it is your view on the subject of discussion. Every opinion is important and should be freely expressed. What we will learn from you today will be useful in the future in strengthening Bilharzia control measures. We wish to request your permission to take notes and to tape record the conversation so as to ensure that we do not miss or misinterpret any of your views after the discussion.

Do you have any questions about the study? YES/NO (**Moderator to record questions and answers provided**)

Thank you for your assistance.

**Bilharzia knowledge and awareness**

**1. Have you ever heard of Bilharzia?**

Probe for:

- Local name.

**2. Where did you first learn about Bilharzia?**

Probe for:

- Newspapers and magazines
- Mass media: Radio; TV; Billboards
- Brochures, posters and other printed materials
- Professionals: Health workers; Teachers
- Family, friends, neighbours and colleagues
- Leaders: At Baraza; Religious leaders
- Other (please explain):

**3. What are the signs and symptoms of Bilharzia?**

**4. How does a person get Bilharzia?**

Probe for:

- Perceived Cause of Bilharzia
- How it spreads

**5. What kinds of people are likely to get Bilharzia?**

Probe for;

- What populations are most likely to contract Bilharzia?
- What are the reasons which make you think so?
- Gender?

**6. How can a person avoid getting Bilharzia?**

Probe for:

- Current interventions by community, government, others.
- What is perceived to have worked in the past and the reasons for such perceptions
- What is perceived to have failed and the reasons for such perceptions

**7. (a) Is Bilharzia curable?**

Probe for:

- How can someone with Bilharzia be cured?
- Herbal remedies
- Home rest without medicine
- Praying
- Specific drugs given by health centre
- Any other method.

**(b)** How effective do you think the different practices you have mentioned of curing Bilharzia are?

Probe for:

- What do you perceive as most effective?
- What are the reasons for this perception?
- What has been less effective?
- What are the reasons they are perceived to be less effective?

**8. What does this community know about Bilharzia control interventions? (**Ensure control measures are mentioned not just cure**)**

Probe for:

- Please list all interventions you are aware of.
- For each intervention listed: What do you understand it to mean, or how do you think it works?
- Understanding of the campaigns.
- Acceptability
- Accessibility / Reach (define)
- Affordability

**9. What would you say about Bilharzia diagnosis and treatment in this region?**

Probe for:

- Acceptability
- Accessibility of treatment
- Affordability- cost
- Effectiveness

**10. How much do you think Bilharzia treatment costs in this country?**

- Exact cost figures (perceived or real)
- Perception of the costs (high, moderate, low)
- How perceived (or real) costs may influence care seeking

**11. In your opinion, how serious a disease is Bilharzia?**

Probe for:

- How serious a problem do you think Bilharzia is in Nyalenda?
- What prompts you to think this way/ please elaborate your answer.

**12. Earlier at the beginning of our discussion you listed some of your sources of information about Bilharzia. Do you feel well informed about Bilharzia?**

Probe for

- What are your other sources of information about Bilharzia?
- How adequate do you think these sources are for information about Bilharzia?
- If you could get more information about Bilharzia, what additional information would you wish to get?
- Preferred source of information.
- Most accessible source. What makes you say this source is more accessible?

**Bilharzia attitudes**

**13. Do you think you can get Bilharzia?**

Probe for: What makes you feel you are either at risk, or not at risk of getting Bilharzia?

**14. What would be your reaction if you found out that you have Bilharzia?**

Probe for and explore why they may have any of these reactions (probe for each):

- Fear
- Surprise
- Shame
- Embarrassment
- Sadness or hopelessness

**15. Who would you talk to about your illness if you had Bilharzia?**

Probe for:

- Doctor or other medical worker
- Spouse
- Parent
- Child(ren)
- Other family member
- Close friend
- No one
- Other:

Probe for:

- What would prompt you to talk about your illness?
- What would make you choose a specific person to talk to about your illness?

**16. Do you know people who have/had Bilharzia?**

**17. In your community, how is a person who has Bilharzia usually regarded/treated?**

**Health- seeking practices**

**18. (a) Where do you usually go if you are sick, or to treat a general health problem?**

Probe for:

- Private clinic
- Government clinic or hospital
- Traditional or homeopathic healer
- Clinic run by nongovernmental organization or church
- Other

**(b)** What prompts people to seek treatment at the places you have mentioned?

**(c)** Are certain care options more likely to be used than others?

**(d)** Under what circumstances does this happen?

**19. How often do you generally seek health care at a clinic or hospital?**

**20. Would you go to the health facility if you suspect you have Bilharzia?**

**21. If you had symptoms of Bilharzia, at what point would you go to the health facility?**

Probe for:

- Low
- Moderate
- severe

**22. If you would not go to the health facility, what is the reason?**

Probe for:

- Not sure where to go
- Cost
- Difficulties with transportation/distance to clinic
- Do not trust medical workers
- Do not like attitude of medical workers
- Cannot leave work (overlapping work hours with medical facility working hours)
- Do not want to find out that something is really wrong
- Pursue other self-treatment options (herbs, etc.)
- Go to pharmacy
- Go to traditional healer

**23. What worries you the most when you think about Bilharzia?**

Thank you very much for participating!!
