# Supplementary material for: Low Levels of Awareness Despite High Prevalence of Schistosomiasis among Communities in Nyalenda Informal Settlement, Kisumu City, Western Kenya
Source: PLoS Negl Trop Dis. 2014 Apr 3;8(4):e2784. doi: 10.1371/journal.pntd.0002784 (PMC3974654; doi:10.1371/journal.pntd.0002784)
Supplement: Text S2 — Key informant interview guide. (DOC) [file pntd.0002784.s003.doc]

**Supporting Text S2: Key Informant Interview Guide**

**Who to Interview: Community Resource Persons (CORPs) and Leaders**

**Introduction:**

Good day. I am … … and am from...... …….. and am here to learn from you about Bilharzia control in this community. The objective of this study is to collect information on the knowledge, attitude and practices on Bilharzia and its control in Nyalenda. The information you provide will be used to improve Bilharzia control. We have invited you because of your experience in this community and the confidence we have in you to be able to reflect the situation in Nyalenda. We will talk to you for about 30-45 minutes. Participation in this discussion is voluntary. Your name and what you say to us during this discussion will be kept confidential.

Are you willing to participate? Yes [ ] No [ ]

**If No, thank respondent and terminate interview**

**Level of implementation of Water, Sanitation and Deworming services.**

1. Is deworming one of the health services provided by the health facility in this community?

2.How satisfied are you with these deworming services? – (probe for extent of satisfaction)

**Priority Health issues**

3. Is Schistosomiasis and other worm infections a priority health problem in this area?

Probe for

- Statistics- level of infection
- Infection groups
- Hospital data

**Interventions/ strategies/ practices for Water, Sanitation and Deworming.**

4. What is being done to address the health problems identified above?

Probe for:

- Methods of intervention
- Intervention for Bilharzia

**Effectiveness of the delivery of water,** **sanitation and deworming interventions**

5. What water, sanitation and deworming activities are ongoing at the moment? (List all the activities)

6. Which areas are covered in each of the activities/ interventions?

7. What group of people are benefiting from each of the interventions?

**Partners and Stakeholders**

8. Apart from the government health facility what other health providers are available in your community?

Probe for:

- The type services provided
- Do they provide water, sanitation and deworming services as well.

**Perceptions and attitudes towards water, sanitation and deworming**

10. What is your opinion about the following:

1. What the government is doing about water, sanitation and deworming services at the community level? Probe for
   - Appropriateness,
   - Relevance to the priority health issues
   - Their attitude to what changes are necessary
2. Water, sanitation and deworming services (formal and informal);

Probe for:

- Affordability,
- Accessibility, reach
- Community participation
- Acceptance
- Relevance of the health services to the prevailing health issues.

.

11. What roles do social groups play in water, sanitation and deworming service delivery?

Probe for:

- Involvement based on gender
- Involvement based on minority groups

**Opportunities, challenges and synergies**

12. What conditions and circumstances can you take advantage of for promoting water, sanitation and deworming of the people in this community?

Probe for:

- Linkages between programmes

13. What obstacles do you face in promoting health services in this community?

14. How are you addressing these obstacles?

Probe for:

- Linkages between programmes
