# Supplementary material for: Low Levels of Awareness Despite High Prevalence of Schistosomiasis among Communities in Nyalenda Informal Settlement, Kisumu City, Western Kenya
Source: PLoS Negl Trop Dis. 2014 Apr 3;8(4):e2784. doi: 10.1371/journal.pntd.0002784 (PMC3974654; doi:10.1371/journal.pntd.0002784)
Supplement: Text S3 — Social demographic profile. (DOC) [file pntd.0002784.s004.doc]

**Supporting Text S3: Social Demographic Profile**

Date of interview: _______________ Assigned Number: ___________

Settlement Unit:_________________

1. Participant’s gender Male [ ] Female [ ]
2. Participant’s age _____
3. What is the participant’s level of education?

None [ ] Primary [ ] Secondary [ ] Post Secondary [ ]

1. Which religious group do you/ name belong to?

Catholic [ ] Legio Maria [ ] Anglican [ ] Baptist Methodist [ ] SDA [ ] Presbyterian [ ] Muslim[ ]

Traditionalist [ ] Roho [ ] Nomiah [ ] No Religion [ ]

Other specify………………………………………………………………………….

1. What is the marital Status?

Divorced [ ]

Living with another [ ]

Married [ ]

Separated [ ]

Single (never married) [ ]

Widowed [ ]

Would rather not say [ ]

1. What is your most important income-generating activity?

Subsistence farming [ ] Fishing [ ] Salaried worker (e.g teacher, medical worker, office….) [ ]

Business owner (e.g duka, kiosk) [ ]

Skilled labour (e.g carpenter, tailor, jua kali) [ ]

Unskilled labour (e.g Shamba, construction)

Other, Specify ……………………………………………………………………

1. What group are you representing?

Volunteer

CHW

Leader of a group

Church leader

TBA

Herbalist

Other Specify [ ]

1. Health Trainings attended (At least 3 most recent)

| Training | Year | Duration |
| --- | --- | --- |
|  |  |  |
|  |  |  |
|  |  |  |
