# Supplementary material for: Low Levels of Awareness Despite High Prevalence of Schistosomiasis among Communities in Nyalenda Informal Settlement, Kisumu City, Western Kenya
Source: PLoS Negl Trop Dis. 2014 Apr 3;8(4):e2784. doi: 10.1371/journal.pntd.0002784 (PMC3974654; doi:10.1371/journal.pntd.0002784)
Supplement: Text S4 — Consent form. (DOC) [file pntd.0002784.s005.doc]

**Supporting Text S4: Consent Form**

**Low Levels of Awareness despite High Prevalence of Schistosomiasis among Communities in Nyalenda Informal Settlement, Kisumu City, Western Kenya.**

**a. Statement that the study involves research**

This project in which we want you participate is a research activity.

**b. Explanation of the purposes of the research**

We would like to understand community knowledge, attitudes and practice towards Bilharzia control in this urban setting in Kisumu. The purpose is to gain information that will eventually help in strengthening the Bilharzia control activities in your community.

**c. Description of the procedures to be followed**

We will ask questions about many aspects of life in this community (household/family). We want to learn more about you and your health care experience. With your permission, we may take pictures of activities in the community as well as the group and take notes, which will help us to explain our findings. We will go to different villages, talk with women and men like you in groups. With your permission, some of the conversations may be tape recorded, so that we do not miss out some of the important things that are said.

**d. Expected duration of participation in the research**

We will talk to you for about 45 -60 minutes. The questions are general but if you find that some questions are not going well with you, please do not feel compelled to answer any of them for any reason.

**e. Disclosure of appropriate alternative to participation**

We shall appreciate your participation in this study; however, feel free to decline this request if you are uncomfortable. Taking part in this study will not cost you or your family anything. You may also leave the study at any time, even after agreeing to participate. You can leave for any reason without any problems.

**f. Description of any benefits to the subject or to others, which may reasonably**

**be expected from the research**

You and your family may not get any direct benefits from being in this study but what you tell us will help us better develop a strategy for strengthening Bilharzia control activities and thus improve the health of the people in this community.

**g. Risk involved**

The risk involved in this research is minimal. The risk relates to possible inconveniences with regards to privacy and confidentiality. However, in a group discussion such as this we ask you to only speak of general community experiences, rather than revealing individuals’ names. We also request that you do not share personal information from this discussion with people in the community.

**h. Confidentiality of records**

Your name and what you say to us for this study will be kept private as much as the law allows. The information you provide shall remain confidential, and your names or any information that may identify you will not be included in reports. The tapes, notes, picture and transcripts shall be stored in a place where only the research team will have access. This will be for a period of one year after completing the study, they will be destroyed.

**i. Questions about research**

1. Do you have any questions about the study and your participation? (Yes/No).
2. Do you agree to participate in this group discussion? (Yes/No)

If you have any questions about this study, you may contact Dr. Pauline Mwinzi at the Kenya Medical Research Institute, Kisumu Tel; 057-2022929 during the study and in the future. If you have concerns about human rights, ethics and welfare issues you may contact the Secretary of the National Ethical Review Board, Kenya Medical Research Institute; Tel; 020-2722541.

If you agree to answer our questions and participate in this study, you can tell us that you agree by repeating these words and then putting your name and signature in the space below.

*I have read (or been read for) the foregoing information, or it has been read to me. I have had the opportunity to ask questions about it and any questions I have asked have been answered to my satisfaction. I consent voluntarily to participate as a subject in this study and understand that I have the right to withdraw from the study at any time without in any way affecting my further health care.*

*_______________________________________* ____________

Individual Participant’s name/ signature Date

______________________________________ ____________

Interpreter/Witness’s signature

Date

_______________________________________ ____________

Person conducting the informed consent signature Date
